# Choline kinase inhibition promotes ER-phagy

**DOI:** 10.1016/j.jlr.2022.100213

**Published:** 2022-04-18

**Authors:** Mahtab Tavasoli, Sandhya Chipurupalli, Christopher R. McMaster

**Affiliations:** Departments of Pharmacology and Biochemistry & Molecular Biology, Atlantic Research Centre, Dalhousie University, Halifax, Nova Scotia, Canada

**Figure:**
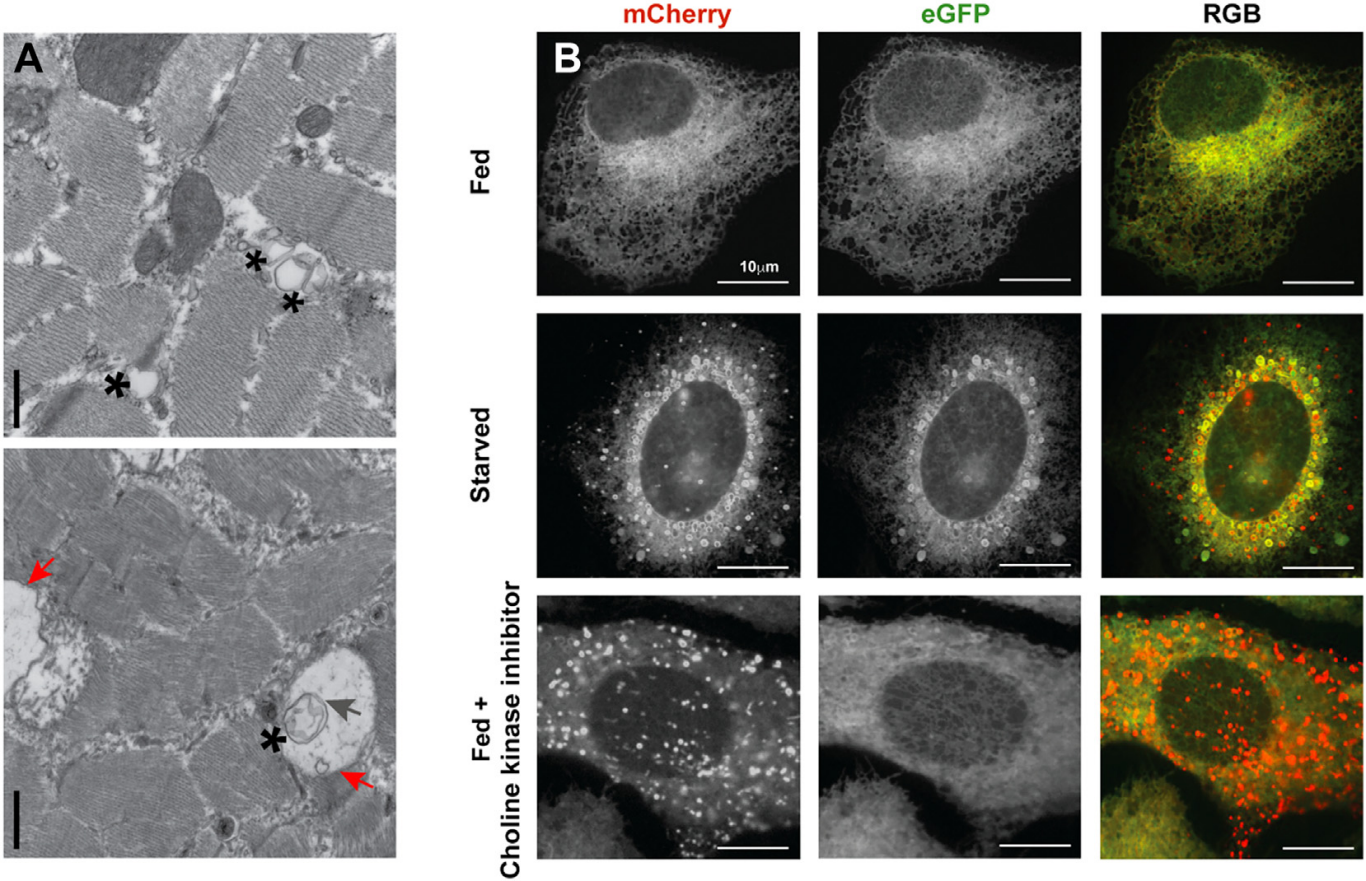

The major membrane phospholipid phosphatidylcholine is predominantly synthesized through the CDP-choline (Kennedy) pathway at the endoplasmic reticulum (ER). The first step in this pathway is the phosphorylation of choline by choline kinase. Humans contain two choline kinase isoforms, *CHKA* and *CHKB*. Variants in the *CHKA* gene that decrease enzymatic activity have been shown to cause an inherited neurodevelopmental disorder with epilepsy, while recessive inheritance of nonfunctional *CHKB* alleles causes an inherited rostrocaudal muscular dystrophy (1, 2). In affected muscle of *Chkb*^*−/−*^ mice, we previously determined that CHKB protein is absent and CHKA expression was severely downregulated, resulting in an almost complete absence of choline kinase activity (2). It is known that stress to the ER activates several ER quality control processes, namely the unfolded protein response, ER-associated degradation, and the relatively newly described ER-phagy. ER-phagy results in engulfment of sections of ER into autophagosomes and transport to lysosomes to aid in replenishment/recycling of damaged ER (3). Panel A: Transmission electron microscopy of *Chkb*^*−/−*^ muscle demonstrates extensive ER injury, including ER vacuolization and expansion (asterisk) and evidence of damaged ER (gray arrow) that is engulfed by autophagosomes and fused with lysosomes (red arrow). These findings are highly suggestive of ER-phagy; the scale bar represents 500 nm. Panel B: Human bone osteosarcoma epithelial cells (U2OS) were treated with the choline kinase inhibitor EB3D (15 μM for 48 h) and ER-phagy monitored using the ER autophagy tandem reporter (EATR) (3). The EATR is a tandem enhanced GFP-mCherry ER-resident protein where both reporters normally face the cytoplasm and fluoresce. In the setting of ER-phagy, mCherry preferentially fluoresces because of the acidic pH of the lysosome (3). As expected, and as previously reported, EATR localizes only to the ER when cells are fed, while under starvation (to induce ER-phagy), mCherry-only puncta were observed (3). The mCherry puncta were also observed when fed cells were treated with the choline kinase inhibitor EB3D. These data suggest that ER-phagy occurs upon inhibition of choline kinase and ER-phagy could play a role in the etiology of diseases associated with choline kinase inhibition.

**EQUIPMENT:** Transmission electron microscope (JEOL JEM-1230; JEOL) with Hamamatsu ORCA-HR digital camera (Hamamatsu) and Zeiss Axio Observer Z.1 Spinning Disk Confocal Microscope (Zeiss).

**REAGENTS:** TetOn-mCherry-enhanced GFP-RAMP4 (EATR) was a gift from Jacob Corn (Addgene; plasmid #109014), Lipofectamine 3000 (Thermo Fisher Scientific), EB3D (MCA® MedChemExpress; catalog no.: HY-115463), doxycycline (Sigma-Aldrich; catalog no.: D9891), and Earle's balanced salt solution (Thermo Fisher Scientific; catalog no.: 24010043).

## Conflict of interest

The authors declare that they have no conflicts of interest with the contents of this article.
